# Excess All‐Cause Mortality by Age and Gender During the COVID‐19 Pandemic in the Federation of Bosnia and Herzegovina, Bosnia and Herzegovina: 2020–2022

**DOI:** 10.1111/irv.70086

**Published:** 2025-02-26

**Authors:** Šeila Cilović‐Lagarija, Johanna Thea Mølgaard Rantzau, Siniša Skočibušić, Sanjin Musa, Armin Sprečo, Amna Isaković, Mirza Palo, Faris Dizdar, Hidajeta Čolović, Veronica Ivey Sawin, Jens Nielsen, Pernille Jorgensen

**Affiliations:** ^1^ Institute for Public Health Federation of Bosnia Herzegovina Sarajevo Bosnia and Herzegovina; ^2^ The EuroMOMO Network Statens Serum Institut Copenhagen Denmark; ^3^ Faculty of Medicine University of Mostar Mostar Bosnia and Herzegovina; ^4^ Regional Executive Office Region Östergötland Linköping Sweden; ^5^ Department of Health, Medicine, and Caring Sciences Linköping University Linköping Sweden; ^6^ World Health Organization, Country Office in Bosnia and Herzegovina Sarajevo Bosnia and Herzegovina; ^7^ Federal Statistical Office Sarajevo Bosnia and Herzegovina; ^8^ World Health Organization, Regional Office for Europe Copenhagen Denmark

**Keywords:** excess mortality, COVID‐19, mortality

## Abstract

**Objectives:**

COVID‐19 has had a profound impact on global mortality and morbidity, yet only a fraction of deaths was confirmed and reported. We estimated all‐cause excess mortality from 1 January 2020 to 31 December 2022 in the Federation of Bosnia and Herzegovina (FBiH) to assess the true magnitude of the pandemic.

**Methods:**

Data for this analysis was sourced from the FBiH mortality register and supplemented with population statistics and official COVID‐19 death counts (i.e., cases where COVID‐19 was registered as the cause of death). Using a Poisson model, all‐cause excess number of deaths and rates per 100,000 person‐years, adjusted for registration delays and stratified by age and gender, were calculated.

**Results:**

FBiH experienced three periods of excess all‐cause mortality throughout the first 3 years of the pandemic, with a total of 12,000 excess deaths, highest among adults 45–74 years and males. No excess mortality was observed in children <15 years.

**Conclusions:**

The true mortality impact of COVID‐19 in FBiH was substantially higher than the reported deaths, including among younger adults. Strengthening civil registration and vital statistics, including establishment of all‐cause mortality surveillance, is essential for improved monitoring of future pandemics and other important public health events. A detailed review of the direct and indirect effects of COVID‐19 on mortality should be conducted to identify areas that require more resources, improve health provision and inform mitigation efforts in future pandemics to save lives.

## 1 Introduction

As of 31 December 2022, the COVID‐19 pandemic has led to a total of 656 million reported cases and 6.7 million COVID‐19 fatalities globally [1].

Accurate measurements of COVID‐19 mortality and the indirect death toll have not only played an important role in predicting the pandemic's progression but have also been of great importance for countries and regions to grasp the full extent of the pandemic's impact on mortality and public health, and for evaluation of policies.

COVID‐19 mortality is an important indicator for monitoring the evolution of SARS‐CoV‐2 transmission and clinical severity of infection. Nevertheless, reported COVID‐19 deaths are subject to testing strategies and capacity, case definitions, standards for coding of deaths, health information system structures and data collection capacities, among others. In addition, it has been widely documented that the pandemic indirectly resulted in an increase of non‐COVID‐19 deaths due to disruption of normal societal activities, overburdened health systems or restricted access to health care [2, 3, 4, 5, 6]. Some studies, however, have reported negative excess mortality during periods of lockdowns in the pandemic which may have been attributed to fewer car accidents, injuries, decline in infectious diseases and reduced air pollution [3, 7]. For these reasons, the concept of all‐cause excess mortality, characterized as the net difference between the number of all‐cause deaths during the pandemic and the expected number of deaths based on historical all‐cause mortality, offers a crucial measure for understanding the true impact of the COVID‐19 pandemic [7, 8]. Furthermore, monitoring of all‐cause mortality, if done in a timely manner, can play a critical role in early warning, impact assessment and in guiding policy decisions and public health actions [9].

Bosnia and Herzegovina,^1^ an upper middle‐income country of around 3.5 million people, experienced its first case of COVID‐19 in March 2020. The initial cases were mainly imported from other European countries [11]. By April 2020, the World Health Organization (WHO) classified Bosnia and Herzegovina as a country with potential for community transmission, prompted by 816 COVID‐19 cases and 35 deaths [12]. By 2023, Bosnia and Herzegovina ranked among the countries with the highest COVID‐19 mortality rates globally [13].

Public health and social measures were implemented in March 2020 by the governments in all entities [11, 14]. These measures encompassed full lockdowns for children and people over 65 years, curfews, border closures, mandatory isolation, mandatory face masks, etc. [14]. These measures were enacted to mitigate further transmission. Within a month, the number of new cases in Bosnia and Herzegovina stabilized. However, when authorities lifted certain measures in the spring and summer of 2020, the number of confirmed cases increased rapidly. In response, selected measures were reintroduced in late summer of 2020 [15].

Vaccination is considered the most effective intervention against SARS‐CoV‐2 infection and serious COVID‐19 illness [16]. Vaccination for COVID‐19 in FBiH was rolled out from early March 2021: first for the elderly (≥75 years of age) and healthcare workers, and from April 2021 for all persons 18 years and above. Revaccination (boosters) was initiated in August 2021 [17].

The aim of this study was to estimate all‐cause weekly excess mortality in the Federation of Bosnia and Herzegovina (FBiH) through the 3 years of the COVID‐19 pandemic (2020–2022), stratified by gender and age, using the EuroMOMO model [17, 18, 19], a model used extensively during the COVID‐19 pandemic for estimating all‐cause excess mortality across Europe.

## 2 Methods

### 2.1 Data Sources

To estimate excess mortality in FBiH during the COVID‐19 pandemic, we used annual retrospective counts of all‐cause deaths by gender and age. Data was obtained from the mortality register of FBiH, which is maintained by the Institute for Statistics of FBiH.

When a person dies in FBiH, a death certificate is filled out by a medical doctor or another healthcare professional. Subsequently, these certificates are gathered for each canton (*N* = 10) in FBiH and recorded in its mortality register. Approximately 70% of the deaths are recorded within the same month as the death occurred, while the remaining 30% are recorded in the succeeding 3 months period. Following data entry, records undergo a thorough review and are updated over a 30‐day period to ensure the accuracy of the information after which they are locked. The final data on deaths is published annually in the statistical yearbook from Institute for Statistic of FBiH [20].

To account for the delay in death registration, we used data through the end of 2023 to ensure that all deaths occurring in 2022, but *registered* in 2023, were included in the analysis. However, we did not include any deaths occurring in 2023 in our analysis.

Information on population size by year, 5‐year age‐groups and gender was extracted from the Institute for Statistics of FBiH database [20]. Weekly population estimates were interpolated by linear interpolation.

In addition, information regarding the officially reported number of COVID‐19 deaths (i.e., cases where COVID‐19 was registered as the cause of death) was obtained from The Institute for Public Health, FBiH [21].

### 2.2 Statistical Methods

For the purpose of this study, we applied the EuroMOMO model, which is a time series Poisson regression model accounting for overdispersion and incorporating trend and seasonality [22]. ISO‐week is used as time unit [23], and the number of weekly deaths is the dependent variable. The primary outputs include the weekly count of all‐cause deaths, the expected weekly count of all‐cause deaths (baseline), the weekly count of excess deaths (calculated as observed minus expected) and the standard deviation around the baseline (represented as z‐score).

The model was applied separately to the age groups, 0–14, 15–44, 45–64, 65–74, 75–84 and 85 + years, as well as for males and females [22]. Differentiated age bands were used to account for variations in disease severity across different age groups to inform targeted public health interventions as well as ensuring sufficient statistical power. Estimates of all‐cause total excess mortality over these strata were pooled, thus adjusting, by stratification, for disparities between genders and age groups. Additionally, we calculated excess mortality per 100,000 person‐years with 95% uncertainty intervals (UI).

All analyses were conducted using the R Statistical Software Version 4.3.1 and the EuroMOMO R‐package MOMO (https://github.com/JensXII/MOMO).

## 3 Results

**FIGURE 1:**
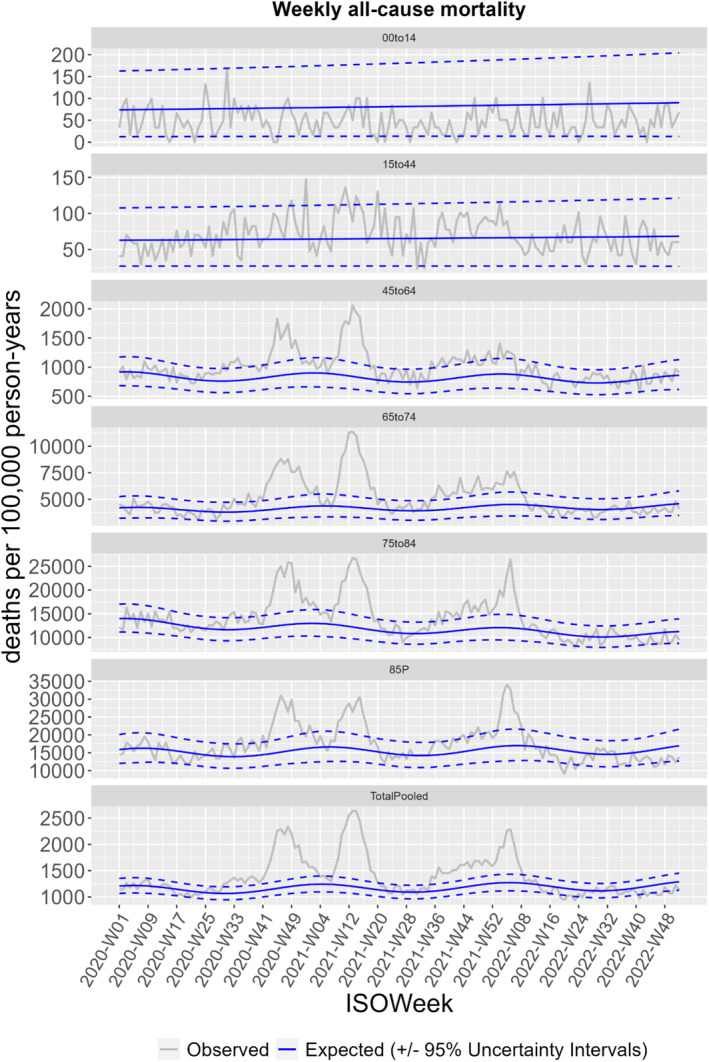
All‐cause mortality per 100,000 person‐years in the Federation of Bosnia and Herzegovina, Bosnia and Herzegovina through the COVID‐19 pandemic, 2020 to 2022.

**FIGURE 2:**
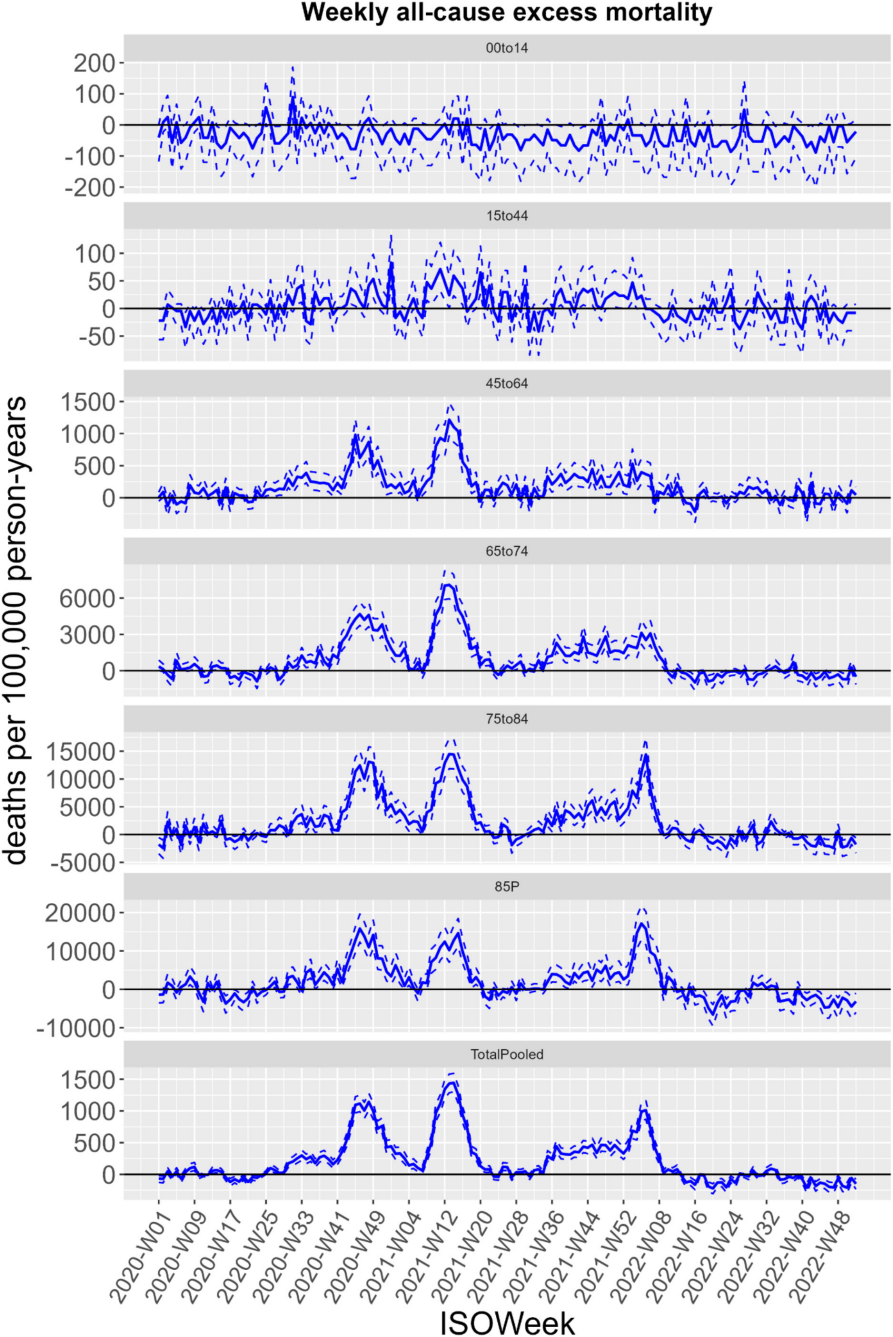
All‐cause excess mortality and 95% Uncertainty Intervals in the Federation of Bosnia and Herzegovina, Bosnia and Herzegovina through the COVID‐19 pandemic, 2020 to 2022.

FBiH experienced three periods of all‐cause excess mortality during the 3 years of the COVID‐19 pandemic. All‐cause mortality in FBiH first exceeded expected levels around Week 28, 2020, and increased substantially to peak between Weeks 44 and 49, 2020 (Figures 1 and 2). Mortality reached expected baseline levels again in February 2021 for a short period. Beginning around Week 7, 2021, until late spring 2021, FBiH experienced a second wave of increased mortality. After a period with relatively low mortality over the summer 2021, FBiH experienced a third wave of mortality from early autumn 2021 through Week 8, 2022, peaking at the very beginning of 2022. From late winter 2022 until the end of 2022, mortality was within normal levels.

In total, in the 3 years of the pandemic, 2020 to 2022, FBiH experienced an estimated 12,067 all‐cause excess deaths (18% above the expected) (Table 1), corresponding to an all‐cause excess mortality of 212.9 (206.7–219.1) deaths per 100,000 person‐years (Table 2). The number of all‐cause excess deaths varied between the years, with 4354 deaths (20%), 7023 deaths (32%) and 690 deaths (2%) in 2020, 2021 and 2022, respectively (Table 1). Corresponding all‐cause excess mortality per 100,000 person‐years in the 3 years was 226.4 (216.0–237.0), 374.1 (361.1–387.1) and 36.9 (30.8–43.4), respectively (Table 2).

**TABLE 1:** Number of all‐cause excess deaths in the Federation of Bosnia and Herzegovina through the COVID‐19 pandemic by gender and year, 2020 to 2022.

Age group	Female			Male			Both genders^a^		
	Observed	Expected	Excess (%)	Observed	Expected	Excess (%)	Observed	Expected	Excess (%)
2020									
0–14	72	118.7	−46.7 (−39.4)	98	124.5	−26.5 (−21.3)	170	243.2	−73.2 (−30.1)
15–44	184	162.5	21.5 (13.2)	425	412.0	13.0 (3.2)	609	574.5	34.5 (6.0)
45–64	1711	1427.7	283.3 (19.8)	3243	2595.1	647.9 (25.0)	4954	4022.8	931.2 (23.1)
65–74	2690	2256.5	433.5 (19.2)	3810	3014.2	795.8 (26.4)	6500	5270.7	1229.3 (23.3)
75–84	4839	4197.1	641.9 (15.3)	4178	3330.9	847.1 (25.4)	9017	7528.0	1489.0 (19.8)
85+	3173	2742.2	430.8 (15.7)	1976	1663.3	312.7 (18.8)	5149	4405.5	743.5 (16.9)
Total^b^	12,669	10904.9	1764.1 (16.2)	13,730	11139.9	2590.1 (23.3)	26,399	22044.7	4354.3 (19.8)
2021									
0–14	56	128.0	−72.0 (−56.2)	78	124.0	−46.0 (−37.1)	134	252.0	−118.0 (−46.8)
15–44	229	145.5	83.5 (57.4)	471	426.5	44.5 (10.4)	700	572.0	128.0 (22.4)
45–64	1891	1365.3	525.7 (38.5)	3327	2469.8	857.2 (34.7)	5218	3835.2	1382.8 (36.1)
65–74	3292	2310.4	981.6 (42.5)	4387	3106.5	1280.5 (41.2)	7679	5417.0	2262.0 (41.8)
75–84	5248	4092.0	1156.0 (28.2)	4420	3201.9	1218.1 (38.0)	9668	7293.9	2374.1 (32.5)
85+	3486	2846.3	639.7 (22.5)	2112	1757.7	354.3 (20.2)	5598	4603.9	994.1 (21.6)
Total^b^	14,202	10887.6	3314.4 (30.4)	14,795	11086.4	3708.6 (33.5)	28,997	21974.0	7023.0 (32.0)
2022									
0–14	71	140.5	−69.5 (−49.5)	75	125.9	−50.9 (−40.4)	146	266.4	−120.4 (−45.2)
15–44	169	132.9	36.1 (27.1)	375	449.8	−74.8 (−16.6)	544	582.8	−38.8 (−6.7)
45–64	1458	1332.2	125.8 (9.4)	2561	2401.7	159.3 (6.6)	4019	3733.9	285.1 (7.6)
65–74	2433	2413.4	19.6 (0.8)	3399	3264.3	134.7 (4.1)	5832	5677.6	154.4 (2.7)
75–84	4230	4070.5	159.5 (3.9)	3390	3142.9	247.1 (7.9)	7620	7213.4	406.6 (5.6)
85+	3061	3012.1	48.9 (1.6)	1849	1894.7	−45.7 (−2.4)	4910	4906.9	3.1 (0.1)
Total^b^	11,422	11101.7	320.3 (2.9)	11,649	11279.3	369.7 (3.3)	23,071	22381.0	690.0 (3.1)
All years (2020–2022)									
0–14	199	387.2	−188.2 (−48.6)	251	374.4	−123.4 (−33.0)	450	761.6	−311.6 (−40.9)
15–44	582	441.0	141.0 (32.0)	1271	1288.3	−17.3 (−1.3)	1853	1729.3	123.7 (7.2)
45–64	5060	4125.3	934.7 (22.7)	9131	7466.6	1664.4 (22.3)	14,191	11591.9	2599.1 (22.4)
65–74	8415	6980.3	1434.7 (20.6)	11,596	9385.0	2211.0 (23.6)	20,011	16365.3	3645.7 (22.3)
75–84	14,317	12359.7	1957.3 (15.8)	11,988	9675.6	2312.4 (23.9)	26,305	22035.3	4269.7 (19.4)
85+	9720	8600.6	1119.4 (13.0)	5937	5315.7	621.3 (11.7)	15,657	13916.3	1740.7 (12.5)
Total^b^	38,293	32894.1	5398.9 (16.4)	40,174	33505.6	6668.4 (19.9)	78,467	66399.7	12067.3 (18.2)

^a^ Stratified by gender.

^b^ Stratified by age‐groups.

**TABLE 2:** All‐cause excess mortality per 100,000 person‐years and 95% uncertainty intervals (UI) in the Federation of Bosnia and Herzegovina through the COVID‐19 pandemic (1 January 2020 to 31 December 2022) by gender and age.

Age group	Female	Male	Both genders^a^
	All‐cause excess mortality (95% UI)	All‐cause excess mortality (95% UI)	All‐cause excess mortality (95% UI)
2020			
0–14	−30.2 (42.4 to −19.4)	−16.1 (−26.0 to −8.0)	−22.9 (−30.6 to −16.0)
15–44	4.9 (1.9–8.6)	2.8 (0.5–6.2)	3.8 (1.8–6.3)
45–64	114.3 (93.9–136.1)	277.4 (246.5–309.6)	193.5 (175.0–212.6)
65–74	614.5 (528.7–704.5)	1326.7 (1190.5–1467.7)	941.8 (863.5–1022.3)
75–84	1815.8 (1563.5–2080.4)	3483.6 (3158.8–3818.7)	2495.6 (2290.7–2706.1)
85+	2317.6 (1988.6–2662.9)	2962.4 (2481.9–3470.4)	2551.1 (2276.3–2836.2)
Total^b^	181.9 (167.9–196.3)	271.6 (256.4–287.2)	226.4 (216.0–237.0)
2021			
0–14	−47.9 (−63.1 to −34.2)	−28.9 (−41.2 to −18.2)	−38.1 (−47.7 to −29.3)
15–44	19.5 (14.2–25.2)	9.9 (5.6–15.0)	14.6 (11.1–18.4)
45–64	217.8 (191.4–245.4)	376.0 (340.7–412.6)	294.7 (272.7–317.3)
65–74	1397.7 (1279.0–1519.7)	2134.7 (1968.1–2305.7)	1737.2 (1636.8–1839.5)
75–84	3147.1 (2843.2–3461.2)	4757.7 (4402.6–5121.9)	3808.7 (3574.2–4048.0)
85+	3369.5 (2984.6–3769.6)	3267.2 (2755.1–3807.6)	3332.3 (3022.4–3652.1)
Total^b^	350.2 (331.9–368.7)	398.3 (380.2–416.8)	374.1 (361.1–387.1)
2022			
0–14	−46.8 (−63.2 to −32.1)	−32.3 (−45.7 to −20.6)	−39.4 (−49.7 to −29.8)
15–44	8.5 (4.7–13.0)	−16.9 (−23.2 to −11.3)	−4.5 (−7.3 to −2.2)
45–64	52.5 (36.2–70.7)	70.3 (50.2–92.4)	61.1 (48.1–75.2)
65–74	27.6 (2.1–67.7)	220.5 (143.3–308.0)	116.6 (76.4–162.1)
75–84	411.5 (265.6–577.3)	904.0 (708.4–1115.0)	615.2 (491.8–747.5)
85+	247.6 (99.1–435.4)	−403.5 (−704.0 to −164.9)	10.1 (−30.9–84.2)
Total^b^	34.0 (25.6–43.2)	39.9 (31.3–49.1)	36.9 (30.8–43.4)
All years (2020–2022)			
0–14	−41.5 (−49.8 to −33.7)	−25.7 (−32.3 to −19.5)	−33.3 (−38.6 to −28.4)
15–44	10.9 (8.4–13.6)	−1.3 (−2.9 to −0.2)	4.7 (3.3–6.2)
45–64	128.3 (115.5–141.6)	241.8 (224.1–260.0)	183.4 (172.6–194.5)
65–74	676.7 (622.7–732.1)	1221.1 (1140.8–1303.1)	927.5 (880.3–975.5)
75–84	1765.9 (1620.8–1915.1)	2993.2 (2817.1–3172.9)	2270.0 (2155.9–2386.1)
85+	1953.4 (1766.8–2146.2)	1898.3 (1649.1–2159.0)	1933.4 (1783.1–2087.7)
Total^b^	188.9 (180.3–197.6)	237.2 (228.4–246.2)	212.9 (206.7–219.1)

^a^ Stratified by gender.

^b^ Stratified by age‐groups.

Overall excess mortality reached almost 20% among adults, aged 45 years or above, mainly due to high excesses in the years 2020 and 2021 (Table 1), where FBiH experienced the highest excess mortality (Table 2). In children, 0–14 years of age, mortality was below the expected levels in all 3 years (Tables 1 and 2).

In both 2020 and 2021, where excess deaths were elevated, males had a higher excess number of deaths compared to females; 23% to 16% in 2020 and 34% to 30% in 2021 (Table 1). However, when mortality returned to baseline levels in 2022, disparities between the genders also normalized.

**FIGURE 3:**
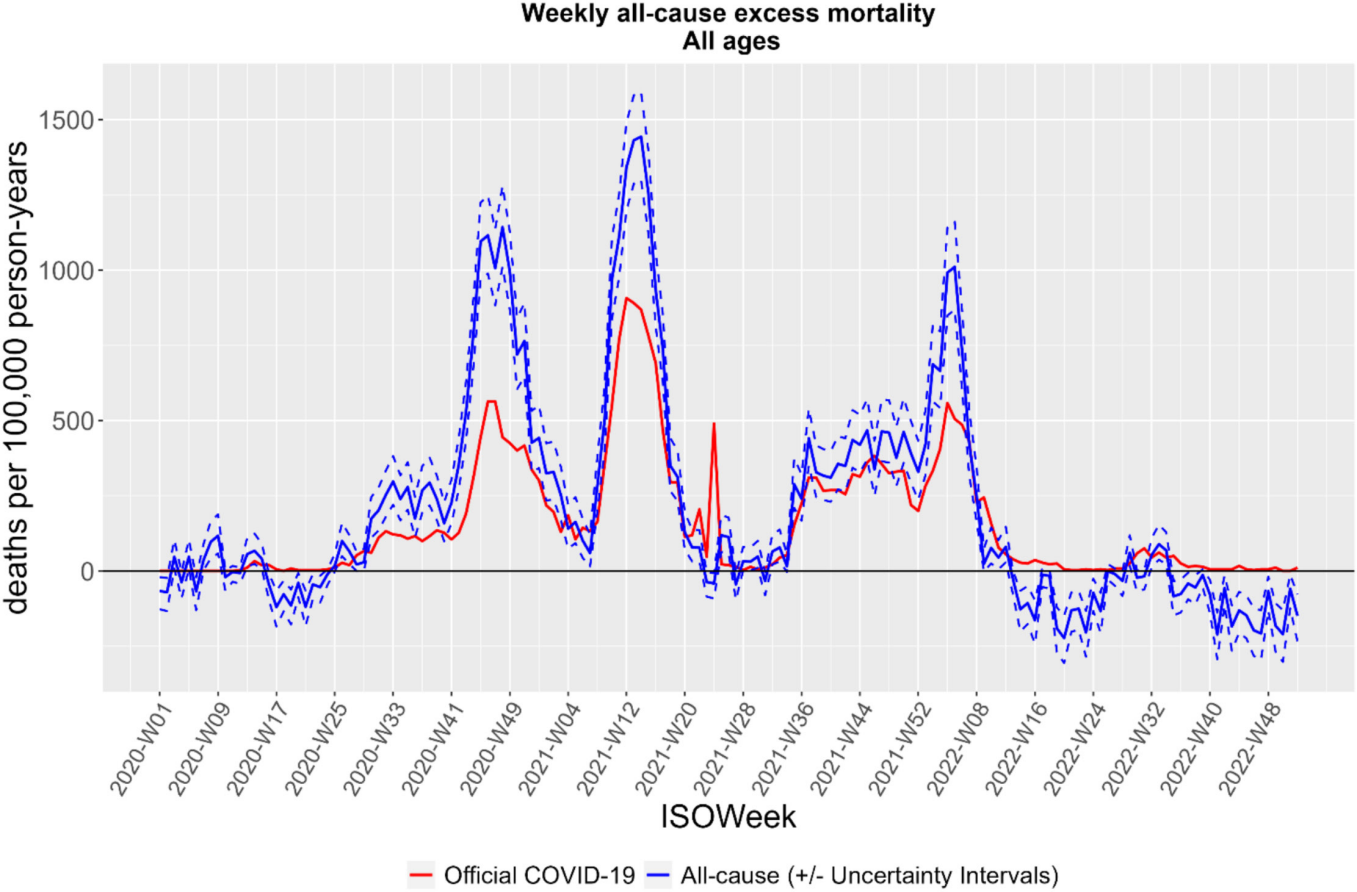
All‐cause‐excess and official* COVID‐19 mortality in the Federation of Bosnia and Herzegovina, Bosnia and Herzegovina through the COVID‐19 pandemic, 2020 to 2022 (*cases where COVID‐19 was registered as the cause of death).

Comparison of all‐cause excess deaths and officially recorded COVID‐19 deaths (deaths attributed to COVID‐19 on the death certificate) for both genders and for all ages (Figure 3) showed that recorded COVID‐19 deaths comprised 51% and 75% of excess deaths in 2020 and 2021 respectively. When mortality levels had normalized in 2022, officially recorded COVID‐19 deaths exceeded all‐cause excess mortality by 36%.

## 4 Discussion

In this study, weekly all‐cause mortality rates by age and gender during the pandemic were estimated in FBiH for the first time. We observed three distinct waves of COVID‐19 through the first three pandemic years; the first in autumn of 2020, the second in spring of 2021 and the third throughout autumn and winter of 2021/2022. The findings showed substantial excess mortality among persons aged 45 years or older, especially among males in 2020 and 2021.

All‐cause excess mortality estimates have provided crucial information on the true health burden of the COVID‐19 pandemic globally, especially because strict lockdowns, international travel restrictions and other mitigations measures virtually eliminated other important causes of seasonal excess death, for example, influenza and other infectious diseases [7].

Unlike many countries in Europe, FBiH did not experience the initial wave of COVID‐19 mortality that reached the continent in spring of 2020 [19]. The promptly enforced and strict public health and social measures implemented from March 2020 [14], before widespread transmission took place, is likely to have played a key role in limiting mortality at this early stage.

As restrictions were relaxed late summer of 2020, mortality began to increase through autumn 2020 and reached a peak by the end of 2020, simultaneously with the circulation of the new SARS‐CoV‐2 variant Alpha [24]. This was followed by another wave of excess mortality in 2021 coinciding with the emergence of the more transmissible Delta variants, not just in FBiH but in most of Europe [23, 25]. With the arrival of the Omicron variant in autumn of 2021 [26], deaths increased again and excess mortality was observed through winter 2021/2022, peaking over the first months of 2022.

Our findings also show increased excess mortality in the age‐group 45–64 years, higher than in other European countries [18]. Throughout the three waves of increased mortality, mainly the population between 45 and 74 years of age were affected. This contrasts findings from other European countries, where the excess mortality was highest among persons above 75 years of age [18], which correlates with other research findings showing that COVID‐19 mortality significantly increased with advanced age during 2020 [27].

Mortality among children, 0–14 years of age, was below expected levels through all the 3 years, a finding also observed in other countries [27, 28]. This may in part be explained by school closures and other preventive measures that drastically reduced or eliminated the circulation of other respiratory pathogens like influenza and RSV [28].

We observed more excess mortality among males than females across age‐groups, a gap that increased during periods with high excess mortality. Increased COVID‐19 mortality among males compared to females have been documented in numerous studies [28, 29, 30]. Yet, the underlying causes of this difference remain unclear. A number of factors may have contributed to these gender‐based disparities in excess mortality in FBiH including differences in population characteristics, for example, higher prevalence among males of certain diseases or factors that increase the risk of the severity of COVID‐19 associated diseases; like hypertension, cardiovascular diseases etc. [31, 32, 33]. Indeed, recent data shows that BiH has one of the highest rates of smoking among adults in Europe, standing at 37.2%, considerably higher than the regional average in the WHO European Region of 24.6% [34]. Additionally, almost half of the male population engages in regular tobacco consumption (45.7%), while roughly one‐third of women do so (28.9%), representing an approximately 12 percentage points difference from the regional norm for both genders [34]. Gender‐based differences in activities and behaviours associated with SARS‐CoV‐2 infection could also, to some extent, explain the higher excess mortality in males if the overall SARS‐CoV‐2 incidence had been greater in this population. Nonetheless, surveys conducted in FBiH during 2020 and 2021 did not suggest any difference in SARS‐CoV‐2 seroprevalence between males and females [35].

The authorities in FBiH implemented public health and social measures encompassing stringent quarantine, indoor mask mandates, physical distancing guidelines, travel restrictions, etc. These measures appear to have played an important role in reducing the transmission of SARS‐CoV‐2 and ensuring that the healthcare system was not overburdened in the beginning of the pandemic, thus initially averting fatalities. Once lifted, circulation of SARS‐CoV‐2 and mortality increased [14].

Deployment of COVID‐19 vaccines has been crucial in diminishing the impact of SARS‐CoV‐2 [36]. As of December 2023, 1.7 billion COVID‐19 vaccine doses had been administered in the European region [37]. However, FBiH achieved relatively low levels of vaccine coverage, with a coverage of 30.8% for the first dose and 29.4% for the second by January 2023 [38]. This could explain the substantial excess mortality FBiH experienced during autumn of 2021, similar to other countries with relatively low COVID‐19 vaccine coverage in the WHO European Region; a finding not observed in countries with high vaccine coverage [39]. These findings highlight the vital role that swift and comprehensive vaccination efforts played in reducing COVID‐19 mortality, especially among risk groups.

However, countries also differ in many aspects related to population survival in a pandemic, such as gross domestic product (GDP), which correlates with the level of healthcare and pandemic response capacity of a country, and has been suggested to be directly related to excess mortality [39]. As expected, wealthier nations possess better resources to cope with public health emergencies like the COVID‐19 pandemic, which could result in lower excess mortality. Studies have also shown a correlation between GDP and vaccination rates, where countries with lower GDPs obtained lower vaccination coverage compared to countries with higher GDPs [37, 38]. Whereas most European countries experienced substantial mortality during the COVID‐19 pandemic, global estimates suggest that excess mortality was generally higher in middle‐income countries in Eastern Europe, including Bosnia and Herzegovina in the first years of the pandemic [13].

In addition, we observed a significant difference between official number of deaths attributed to COVID‐19 compared to all‐cause excess number of deaths in 2020 and 2021, an observation made in most countries globally [40, 41]. This ‘inconsistency’ can be explained by a number of factors. First, assigning a death due to SARS‐CoV‐2 was challenging in persons with multiple co‐morbidities and atypical presentation of COVID‐19 symptoms. Second, FBiH required a SARS‐CoV‐2 test to list COVID‐19 as a cause of death, potentially resulting in missed cases due to false‐negative tests. Third, limited testing capacity in the beginning of pandemic, or lack of testing of those not suspected to have COVID‐19, may also have contributed to the underestimation. Finally, excess deaths due to other causes may be indirectly attributed to the COVID‐19 pandemic as a result of disruptions and delay in health service provisions. In 2022, as mortality normalised, negative all‐cause mortality was observed, which may be due to premature fatalities over the preceding 2 years.

### 4.1 Strengths and Limitations

One factor contributing to the reliability of our results is the minimal circulation of seasonal influenza and other respiratory viruses during the pandemic, reducing the likelihood of misclassifying influenza as COVID‐19. This, along with the absence of significant concurrent public health events during the study period, suggests that the estimated all‐cause excess mortality in FBiH could largely be attributed to the COVID‐19 pandemic. This encompasses both deaths directly associated with COVID‐19 infection and those indirectly linked to the COVID‐19 pandemic due to e.g., delayed access to healthcare for other ailments, as well as various other factors. However, this study also has some limitations. The estimation of expected mortality was based on data preceding the pandemic (2016–2019) and projected throughout the three pandemic years. This may have affected the accuracy of the estimated expected mortality increasingly through the pandemic, for example, by prolonging the linear trend for demographic and structural changes which may, depending on the slope, over‐ or underestimate the excess. It is likely that this effect contributed to the increasingly reduced mortality among children. Second, interruptions or disruptions in the coroner service could have led to significant delays in registration of deaths, potentially impacting the weekly excess mortality estimates.

## 5 Conclusion

The Federation of Bosnia and Herzegovina experienced three significant waves of increased mortality between 2020 and 2022 resulting in 12,000 (18%) excess deaths. Total excess deaths were greater than the recorded number of COVID‐19 deaths highlighting the importance of measuring all‐cause deaths for a precise assessment of the true impact of the COVID‐19 pandemic. Understanding the full scale of COVID‐19‐related mortality in FBiH is essential, not only to gain insights on clinical management of severe SARS‐CoV‐2 cases, but also to address gaps in the capacity of the health system. Further investigations will be needed to quantify the proportion of excess deaths directly caused by SARS‐CoV‐2 infection and those caused indirectly as a result of disruption of health services and society in general.

Monitoring all‐cause excess mortality using the EuroMOMO model could be an important future tool in FBiH for assessing the importance of key public health events including seasonal influenza epidemics, evolution of the COVID‐19 pandemic, new pandemics and extreme weather events in order to inform policies, clinical care, vaccination strategies, resource allocation and preparedness.

## Author Contributions

C.L., J.N., S.M., S.S., M.P. and P.J. conceived the study on which this analysis is based; S.C.L., A.I., J.N., F.D., H.C. and A.S. planned and implemented the study, including development of analysis plan, data quality checks and acquisition of data; S.C.L., M.P., V.I.S., J.N. and P.J. conceived the article; S.C.L., J.T.M.R. and V.I.S. drafted the manuscript and performed the literature search. J.N. performed the data analysis, and F.D. contributed to the data analysis. All authors contributed to the interpretation of the results and critically revised the manuscript. All authors had full access to all the data reported in the study and accept responsibility to submit the paper for publication.

## Ethics Statement

The authors have nothing to report.

## Conflicts of Interest

The authors declare no conflicts of interest.

### Peer Review

The peer review history for this article is available at https://www.webofscience.com/api/gateway/wos/peer‐review/10.1111/irv.70086.

## Disclaimer

P. Mirza, F. Dizdar and P. Jorgensen work for the World Health Organization (WHO). The authors alone are responsible for the views expressed in this publication and they do not necessarily represent the decisions, policies or views of the WHO.

## Consent

Not applicable.

## Data Availability

The datasets used and/or analysed during the current study are available from the corresponding author on reasonable request.

## Endnotes

^1^
Administratively, Bosnia and Herzegovina is comprised of two entities: the Federation of Bosnia and Herzegovina (FBiH) and the Republika Srpska (RS), as well as the Brčko District of Bosnia and Herzegovina [10]. Healthcare administration in Bosnia and Herzegovina is decentralized to entity and district level.
